# *Salmonella* Detection in Food Using a HEK-hTLR5 Reporter Cell-Based Sensor

**DOI:** 10.3390/bios14090444

**Published:** 2024-09-18

**Authors:** Esma Eser, Victoria A. Felton, Rishi Drolia, Arun K. Bhunia

**Affiliations:** 1 Molecular Food Microbiology Laboratory, Department of Food Science, Purdue University, West Lafayette, IN 47907, USA; esmaeser@comu.edu.tr; 2 Department of Food Engineering, Faculty of Engineering, Canakkale Onsekiz Mart University, Canakkale 17100, Turkey; 3 Molecular and Cellular Microbiology Laboratory, Department of Biological Science, Old Dominion University, Norfolk, VA 23529, USA; vfelt001@odu.edu; 4 Purdue Institute of Inflammation, Immunology and Infectious Disease, Purdue University, West Lafayette, IN 47907, USA; 5 Center for Bioelectronics, Old Dominion University, Norfolk, VA 23508, USA; 6 Department of Comparative Pathobiology, Purdue University, West Lafayette, IN 47907, USA

**Keywords:** *Salmonella*, cell-based sensor, HEK-dual hTLR 5, detection, flagella, immunomagnetic separation, food safety

## Abstract

The development of a rapid, sensitive, specific method for detecting foodborne pathogens is paramount for supplying safe food to enhance public health safety. Despite the significant improvement in pathogen detection methods, key issues are still associated with rapid methods, such as distinguishing living cells from dead, the pathogenic potential or health risk of the analyte at the time of consumption, the detection limit, and the sample-to-result. Mammalian cell-based assays analyze pathogens’ interaction with host cells and are responsive only to live pathogens or active toxins. In this study, a human embryonic kidney (HEK293) cell line expressing Toll-Like Receptor 5 (TLR-5) and chromogenic reporter system (HEK dual hTLR5) was used for the detection of viable *Salmonella* in a 96-well tissue culture plate. This cell line responds to low concentrations of TLR5 agonist flagellin. Stimulation of TLR5 ligand activates nuclear factor-kB (NF-κB)—linked alkaline phosphatase (AP-1) signaling cascade inducing the production of secreted embryonic alkaline phosphatase (SEAP). With the addition of a ρ-nitrophenyl phosphate as a substrate, a colored end product representing a positive signal is quantified. The assay’s specificity was validated with the top 20 *Salmonella enterica* serovars and 19 non-*Salmonella* spp. The performance of the assay was also validated with spiked food samples. The total detection time (sample-to-result), including shortened pre-enrichment (4 h) and selective enrichment (4 h) steps with artificially inoculated outbreak-implicated food samples (chicken, peanut kernel, peanut butter, black pepper, mayonnaise, and peach), was 15 h when inoculated at 1–100 CFU/25 g sample. These results show the potential of HEK-Dual^TM^ hTLR5 cell-based functional biosensors for the rapid screening of *Salmonella*.

## 1. Introduction

*Salmonella* is a member of the Enterobacteriaceae family and is a Gram-negative, non-spore-forming motile bacillus with peritrichous flagella [1]. Salmonellae are widespread in the environment and found in various foods and ingredients, posing serious problems to the food industry [2,3,4,5,6]. Non-typhoidal *Salmonella* (NTS) infection is the second most common zoonotic disease after campylobacteriosis in the European Union member states, which reported over 91,000 cases in 2018. According to the Centers for Disease Control and Prevention (CDC), *Salmonella* causes about 1.35 million infections, 26,500 hospitalizations, and 420 deaths annually in the United States [7]. *Salmonella enterica* outbreaks have been associated with various foods, including spices, nuts, flours, bakery products, fresh produce, and poultry, due to their robust physiological adaptability in harsh conditions [8,9].

Noteworthy outbreaks have been reported for *Salmonella enterica* serovar Oranienburg (*S.* Oranienburg) involving black pepper [10] and onion [11,12]; *S.* Montevideo in black and red peppers [13]; *S.* Mbandaka, *S.* Stanley and *S.* Newport in peanuts and peanut butter [14,15]; *S.* Agona, *S.* Anatum, *S.* Braenderup, *S.* Dessau, *S.* Hartford, *S.* Meleagridis, *S.* Muenchen, *S.* Rodepoort, *S.* Tennessee, and *S.* Tornow in shelled peanuts [16]; *S.* Enteritidis in fresh peaches [17], *S.* Heidelberg, *S.* Infantis, and *S.* Tennessee in chicken [18,19,20]; and *S.* Typhimurium definitive type 49 in eggs and mayonnaise prepared with eggs [21], and *S.* Indiana associated with egg mayonnaise sandwiches [21,22]. Moreover, in Brazil, potato salad made with homemade mayonnaise was associated with *S.* Enteritidis [23], and buffet dishes containing mayonnaise were associated with an *S.* Enteritidis outbreak in Germany [24].

Numerous rapid methods, including miniaturized biochemical tests, physicochemical methods that measure bacterial metabolites, nucleic acid-based tests, bacteriophage-based sensors, and antibody-based methods, have been developed [25,26,27]. Conventional methods for detecting *Salmonella* include pre-enrichment, selective enrichment, selective and differential plating, biochemical phenotyping of suspect colonies, and serotyping [25]. These procedures are time-consuming, laborious, and may take five to seven days [28,29,30,31,32]. For consumer protection, risk-based rapid methods with high specificity and sensitivity are necessary to provide confirmed results in a day, and the quality of these results should at least be as reliable as the reference methods [33]. In addition to these approaches, surface-enhanced Raman scattering (SERS) sensing platforms offer exceptional sensitivity and selectivity for detecting trace amounts of contaminants and pathogens in food with rapid, non-destructive analysis. The novelty of SERS lies in its use of advanced nanostructured substrates and the development of portable, real-time devices that facilitate on-site testing and enhance food safety monitoring [34,35].

Despite these developments, there is an increasing interest in the applications of biosensors that incorporate mammalian cells to detect foodborne pathogens to differentiate viable from non-living cells or toxins, which is a critical determinant for the food industry since nonviable pathogens are not considered a threat [32,36,37,38]. Cell-based biosensors (CBBs) have been applied to several detection systems and continue to serve as a reliable method to probe for the presence of pathogens in clinical, environmental, or food samples [39,40]. Mammalian CBBs exploit host–pathogen interactions, including pathogen adhesion, activation of host cell signaling events, cell-cycle arrest, apoptosis, and/or cytotoxicity [32,38]. The ability to detect host–pathogen interaction makes CBB a functional test, thus setting it apart from conventional methods. Most CBBs measure the optical properties of cellular metabolites or intracellular enzymes released after the pathogens’ interaction with mammalian cells. Cytotoxicity assays have also been developed independently to detect *Salmonella* spp., using the Caco-2 cell line [41], RAW264.7 [42], and J774 macrophage cell lines [43]. However, due to the complex nature of food matrices, their specificity or sensitivity cannot be guaranteed only based on cytotoxicity analysis. Alternatively, an analyte-specific amplified cell signaling cascade approach could be adopted to improve the specificity and sensitivity of CBB.

**Figure 1:**
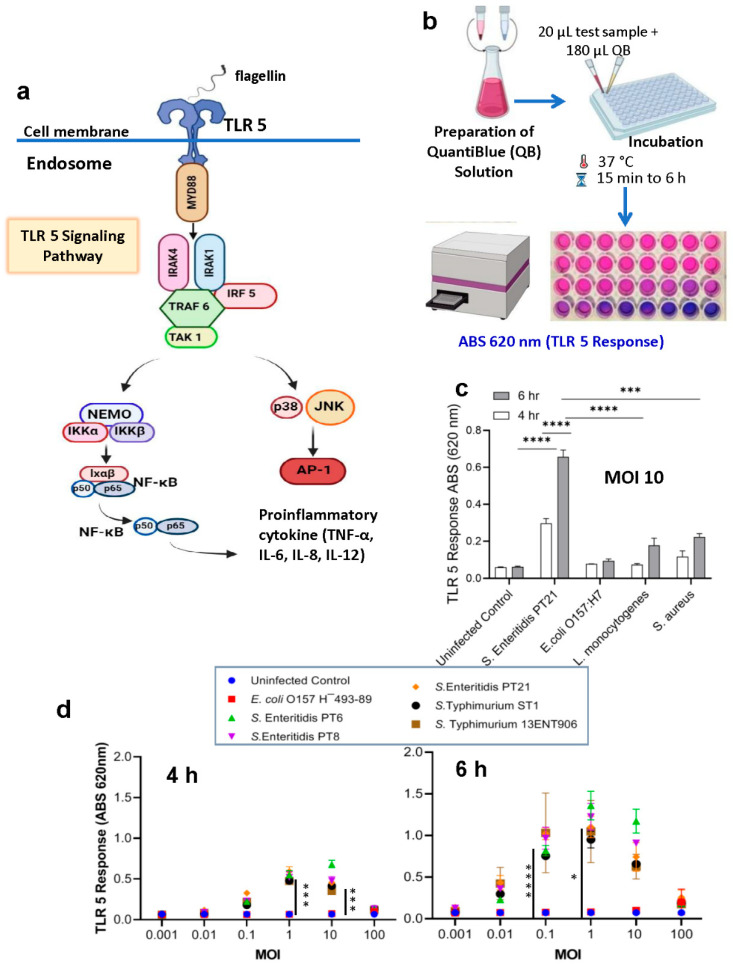
Cell-based assay for detection of *Salmonella* using HEK dual TLR5 sensor. (**a**,**b**) Schematic representation: this panel illustrates the flow diagram of the cell-based assay. The process begins by preparing the Quantiblue solution to track the TLR5 response. HEK cells engineered to express the TLR5 response are then exposed to varying concentrations of *Salmonella bacteria*, leading to an observable color change. The schematic details the steps in the assay, including cell culture, interaction with the bacteria, and the TLR5 response mechanism triggered by the bacterial flagella. Image created using Biorender.com. (**c**) This panel represents the analysis of the HEK dual TLR5 sensor’s response to various foodborne bacterial pathogens tested at MOI of 1 for 4 h and 6 h. (**d**) These panels present the analysis of the signal response from the HEK dual TLR5 sensor cells at different multiplicities of infection (MOI), examining the ratio of HEK cells to bacteria. The MOI ratios analyzed include various levels to determine the sensitivity and dynamic range of the assay. The left panel shows the signal response after 4 h of incubation, while the right panel depicts the response after 6 h. Data in (**c**,**d**) represent the average results from three independent experiments presented as mean ± SEM. *, *p* < 0.05; ***, *p* < 0.0001; ****, *p* < 0.00001.

In this study, our goal was to use an engineered human embryonic kidney (HEK293) cell line expressing Toll-Like Receptor 5 (TLR5) and a chromogenic reporter system (HEK dual hTLR5) as the sensor platform. This cell line responds to low concentrations of TLR5 agonist flagellin [44,45]. Stimulation of TLR5 ligand activates a canonical nuclear factor-kB (NF-κB)-linked alkaline phosphatase (AP-1) signaling cascade, including the production of SEAP (secreted embryonic alkaline phosphatase) (Figure 1a). With the addition of ρ-nitrophenyl phosphate as a substrate, a colored end product representing a positive signal can be detected by the naked eye or quantified spectrophotometrically. Though not all bacterial flagellin are, *Salmonella* flagellin is strongly recognized by TLR5 [46]. In addition, immunomagnetic separation (IMS) systems using anti-*Salmonella* antibody-coated Dynabeads (Invitrogen) can provide specificity by separating magnetic bead-bound *Salmonella* from the sample matrix. The IMS can improve assay specificity and sensitivity by capturing and concentrating target pathogens from complex food matrices before exposure to HEK dual hTLR5 cells [47,48,49].

Overall, the data showed that the HEK dual hTLR5 cell-based sensor could detect viable ~50–100 *S.* Enteritidis cells in spiked food samples (black pepper, chicken, mayonnaise, peanut kernel, peanut butter, and peach) in 15 h (including enrichment steps) with high accuracy and specificity. Our results suggest the potential application of HEK-Dual^TM^ hTLR5 cell-based functional biosensors for the rapid detection of *Salmonella*.

## 2. Materials and Methods

### 2.1. Bacterial Cultures and Motility Testing

Bacterial strains (Table 1) were stored as 10% glycerol stocks at −80 °C [50]. To recover from frozen stock, each strain was streaked onto a tryptic soy agar (TSA) (Thermo Fisher Scientific, Rochester, NY, USA) plate and incubated at 37 °C for 18 h to obtain pure colonies. A single colony of each strain was inoculated and propagated in tryptic soy broth (TSB) with 0.5% yeast (TSBYE; Thermo Fisher Scientific) at 37 °C for 18 h with shaking at 120 rpm.

For the bacterial motility (swimming vs. swarming) assay, a previous method [51] was followed. Briefly, bacteria were spot inoculated onto a TSA plate containing either 0.3% *w*/*v* (for swimming motility) or 0.5%, 0.7%, and 1%, and 1.0% *w*/*v* (swarming motility) agar, incubated at 37 °C for 18–24 h, and their colony diameter was recorded.

### 2.2. Cell Culture

HEK-Dual hTLR 5 (NF-KB-IL8) cells were purchased from InVivoGen (San Diego, CA, USA) and grown in Dulbecco’s Modified Eagle Medium (DMEM) containing heat-inactivated 10% fetal bovine serum with antibiotics (Penicillin-Streptomycin, Normocin, Hygromycin B Gold and Zeocin). Cells were incubated at 37 °C with 5% CO_2_ in a humidified incubator. Cells were seeded in tissue culture flasks, and the medium was refreshed twice a week until cells reached the desired confluency. The HEK 293 hTLR 5 cell monolayer was maintained and harvested using trypsin, and a hemocytometer with Trypan blue staining determined viable cell concentrations. A cell concentration of ~5 × 10^5^/mL was added to each well of a 96-well cell culture plate and incubated overnight at 37 °C under 5% CO_2_ in a humidified incubator for 24 h. Then, HEK293 cells were treated with bacteria to obtain the desired multiplicity of infection (MOI) value.

### 2.3. Cell-Based Biosensor for Detection of Bacterial Cultures

The HEK Dual hTLR 5 cell monolayers were prepared and maintained as described above in a 96-well plate. Overnight-grown (18 h) bacterial cultures were obtained and diluted to the desired concentration using PBS, which was then transferred to the DMEM medium. Heat-killed bacteria suspensions were prepared with heat treatment at 80 °C for 10 min. Heat inactivation of all bacterial cultures was confirmed by plating on tryptic soy agar (TSA) (Thermo Fisher Scientific, Rochester, NY, USA) plates. A 100 µL aliquot of culture suspension in DMEM was added to each well. After 4 and 6 h of incubation at 37 °C, a 20 µL aliquot of the supernatant was collected from each well and transferred into another empty 96-well plate and mixed with 180 µL of the Quanti Blue Solution (InVivoGen, San Diego, CA, USA) used as a detection reagent to quantify SEAP activity. After one hour of incubation at room temperature in the dark, absorbance at 620 nm was measured using a microplate spectrophotometer (BioTech Epoch). SEAP is a truncated form of placental AP and is widely used as a reporter gene. SEAP is secreted into cell culture supernatant and offers many advantages over intracellular reporters. It allows the determination of reporter activity without disturbing the cell monolayers, does not require the preparation of cell lysates, and can be used for kinetic studies.

### 2.4. Specificity/Selectivity of Cell-Based Sensor

HEK Dual hTLR 5 (NF/IL8) reporter cells were generated from HEK-Dual Null cells by stable transfection of the human TLR5 (hTLR5) gene (InVivoGen, San Diego, CA, USA). They respond to low concentrations of TLR5 agonist flagellin. To test the specificity and selectivity of this cell line, a non-motile *E. coli* O157:H¯493-89 and several non-*Salmonella* cultures were used (Table 1).

### 2.5. Limit of Detection of Cell-Based Sensor

The HEK Dual hTLR 5 (NF/IL8) reporter cell line was used to detect bacterial flagellin. NTS *Salmonellae* express flagellin and are motile. To determine the sensor’s detection limit, we prepared *Salmonella* cultures at different dilution levels. The MOI was adjusted to 100, 10, 1, 0.1, 0.01, and 0.001 (HEK cell count was seeded to ~10^5^ bacteria/well). After treating HEK Cells with specified bacteria levels for 4 h and 6 h, each well’s SEAP level was assayed by measuring the absorbance values at 620 nm. The lowest concentration of bacteria, which caused a significant TLR 5 response change, was considered the limit of detection for the sensor. Depending on the experimental design, in most cases an absorbance (620 nm) cut-off value was set at 0.5.

### 2.6. Salmonella Enteritidis Analysis in Spiked Food Samples

Twenty-five grams of each food sample (except peach, which was sampled at 100 ± 5 g) was artificially contaminated with 500 µL of *S.* Enteritidis PT21 suspension and placed in sterile sampling bags in a biosafety cabinet. The contamination level was set to approximately 1 × 10^2^ CFU/g. All samples were mixed with 225 mL (peach—150 mL) of buffered peptone water and incubated at 37 °C for 4 h. Then, 1 mL of pre-enriched sample was transferred to 10 mL of RV broth for selective enrichment and incubated at 42 °C for 4 h.

Following selective enrichment, 1 mL of the enriched sample was mixed with 20 µL of Anti-*Salmonella* Dynabeads (Dynabeads™ anti-*Salmonella* Catalog number: 71002; Thermo Fisher, Rochester, NY, USA) and incubated at room temperature, with gentle agitation for 15–30 min. After incubation, the magnetic beads were separated from the sample matrix using a magnetic separator, and the supernatant was discarded. Beads were washed once with sterile PBS and resuspended in 1 mL PBS, serially diluted, and plated onto TSA and XLD (Xylose Lysisne Deoxycholate; ThermoFisher) plates for enumeration. Plating was performed after each step to determine the microbial load.

RV-enriched bacterial samples with or without IMS were suspended in 1 mL DMEM for cell-based analysis. A total of 100 µL of sample/well was transferred to the HEK cell monolayer in 96-well plates. TLR 5 response analyses were performed following 4 and 6 h of bacteria–cell interaction.

### 2.7. Detection of Stressed Cells

*S.* Enteritidis PT21 cells were freshly prepared in TSB and then exposed to cold (4 °C), heat (45 °C), acid (pH 5.5), ethanol (5%), NaCl (5.5%), and oxidative stress (15 mmol H_2_O_2_) for 3 h [52]. Bacterial cells were washed with PBS, resuspended in DMEM media, added to the HEK cell monolayer, and assayed as above.

### 2.8. Cytotoxicity Assays

To determine HEK cells’ viability during pathogen exposure, supernatants from the wells were assayed for lactate dehydrogenase release (Cayman, Ann Arbor, MI, USA). Two controls were included for calculating percent cytotoxicity (LDH release). The low control consisted of supernatant from untreated cells with no exposure to bacteria. The high control was from cells treated with 0.1% Triton X-100 for one minute. For the plate-based bacterial motility assay, a previous method [51] was followed.

### 2.9. Polymerase Chain Reaction (PCR) Confirmation

Black pepper, chicken thighs, mayonnaise, peanut kernel, peanut butter, and peach samples were inoculated with approximately 1 × 10^2^ CFU/25 g of *S.* Enteritidis PT21 and were placed in 37 °C for 4 h in pre-enrichment media (Buffered Peptone Water). After 4 h of pre-enrichment, samples were enriched selectively in Rappaport-Vassiliadis (RV) broth at 42 °C for 4 h. Then, samples were subjected to testing using the TLR cell-based sensor platform. In parallel, the same set of samples was tested with plate-counting methods. PCR was performed to verify the accuracy of both detection methods. DNA was extracted from the enriched samples using boiling methods.

A PCR reaction was used to amplify the gene targeting the *Salmonella* Invasion protein A (*invA*) with an amplicon size of 796 bp [36]. The forward primer sequence used was 5′CGGTGGTTTTAAGCGTACTCTT3′, and the reverse primer sequence used was 5′CGAATATGCTCCACAAGGTTA3′. The reaction mixture (25 μL) contained 1 μg of DNA template, 0.2 μM of each primer, 2.5 mM MgCl_2_, 200 μM of dNTP, 1× GoTaq Flexi buffer, and 1 U of GoTaq Flexi DNA polymerase (Promega). The reaction took place in the Profelx PCR system with an initial denaturation at 94 °C for 3 min, 35 amplification cycles consisting of 1 min of denaturation at 94 °C, 1.5 min of annealing at 50, and 1.5 min of elongation at 72 °C. DNA amplicons (*invA* 796 bp) were detected in agarose gel (1.5%, wt/vol) containing 1 μg of ethidium bromide/mL [36].

### 2.10. Statistical Analysis

All data were analyzed through GraphPad Prism software (Version 9). An unpaired *t*-test was used when comparing two datasets. ANOVA with Tukey’s multiple comparison test was used to compare more than two datasets. At least 3 independent experiments were performed for all data presented with mean ± standard error of the mean (SEM).

## 3. Results

### 3.1. Optimization of HEK Dual hTLR5 Sensor for Detection of Salmonella

HEK-Blue-hTLR5 cells (InvivoGen, San Diego, CA, USA) were constructed with the human TLR5 gene and an inducible secreted embryonic alkaline phosphatase (SEAP) reporter gene (Figure 1a). The SEAP gene was placed under the control of the IFNβ minimal promoter fused to five NF-κB and AP-1-binding sites. Stimulation with a TLR5 ligand activates NF-κB and AP-1, which induce the production of SEAP. The amount of SEAP production can be determined qualitatively with the naked eye or quantified with the HEK-Blue Detection system (Quanti Blue substrate) by measuring absorbance at 620–655 nm (Figure 1b). Initial experiments were conducted to determine if HEK-Blue-hTLR5 cells are responsive to *Salmonella*. At multiplicity of infection (MOI) 10, HEK cells exposed to *Salmonella enterica* ser Enteritidis (PT21) for 6 h showed a significantly higher response (~6-fold increase; *p* < 0.0001) than *Escherichia coli* O157:H7, *Listeria monocytogenes*, and *Staphylococcus aureus* (Figure 1c). These data indicate that HEK-Blue-hTLR5 can be used to detect *Salmonella* in a cell-based sensor. Bacterial motility assay results on agar plates at different concentrations [51,53] confirmed *S.* Enteritidis to be highly motile, and the most evident colony diameter differences were observed at 0.3% agar (swim plates) compared to the other bacteria tested (Figure S1). Significantly higher flagellar expression in *Salmonella* than other bacterial genera tested may contribute to the observed high signal with the HEK dual hTLR5 sensor than the other pathogens (Figure 1c).

The HEK-hTLR5 Reporter Cell-based Sensor is designed to specifically detect flagellin, a protein component of the flagella of motile bacteria. While our primary focus in this study was detecting *Salmonella* spp., we tested the sensor with other bacteria, such as *Escherichia coli* and *Listeria monocytogenes (*Figure 1c). However, *Salmonella* consistently elicited a higher response, highlighting the sensor’s excellent capacity to detect *Salmonella* specifically. This specificity is likely due to the unique interaction between *Salmonella* flagellin and TLR5. This observation aligns with previous studies reporting that not all bacterial flagellin are, but *Salmonella* flagellin is, strongly recognized by TLR5 [46].

Next, we determined the optimal *Salmonella* cells to HEK cell ratio (multiplicity of infection, MOI) needed to show a positive response after 4 or 6 h of incubation. At 4 h (Figure 1d), MOI 1 and 10 showed 6.7- and 8.7-fold higher signals than the uninfected controls, while at 6 h (Figure 1d), MOI 0.1, 1, and 10 showed 14.2-, 15.3-, and 10.3-fold higher signals, respectively. *E. coli* O157: H¯493-89, a non-motile strain, was used as a negative control. These data indicate that an MOI of 1 exhibits a positive signal after 4 h of exposure, while an MOI of 0.1 is sufficient after 6 h of exposure. However, the TLR-5 response to MOI 0.1 compared to MOI 1 at 6 h did not differ significantly. Therefore, we used MOI 1 (4 h and 6 h) for further experiments.

### 3.2. Specificity of HEK Dual TLR5 Sensor for Detection of Salmonella Serovars

**Figure 2:**
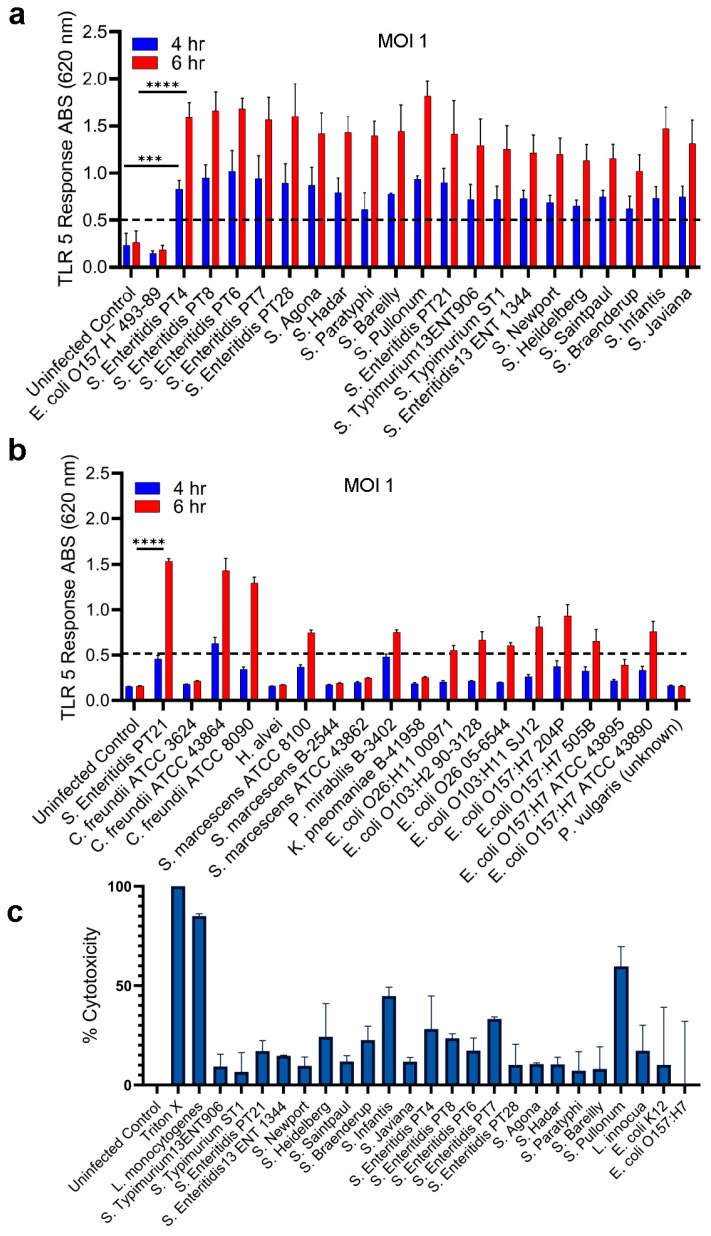
Specificity analysis of the HEK dual TLR5 sensor’s response to various *Salmonella* serovars. (**a**) Specificity analysis of the HEK dual TLR5 sensor’s response to various *Salmonella* serovars. The assay was conducted at a multiplicity of infection (MOI) of 1, with 4 and 6 h exposure times. The data illustrate the sensor’s ability to detect different *Salmonella* serovars specifically. *E. coli* O157:H¯493-89, a non-motile strain, was used as a negative control. (**b**) Specificity analysis of the HEK dual TLR5 sensor’s response against non-*Salmonella* bacteria: this panel shows the analysis of the HEK dual TLR5 sensor’s response to a range of non-*Salmonella* bacteria. Like panel (**a**), the assay was performed at an MOI of 1, with signal responses measured after 4 and 6 h of exposure. *Salmonella* PT21 was used as a positive control. (**c**) Cytotoxicity was determined by using an LDH release assay. The assay was conducted at a multiplicity of infection (MOI) of 1, with 6-h exposure times. A 1% Triton-X100 was used as a positive control (100% cytotoxicity). Data in (**a**–**c**) represent the average results from three independent experiments presented as mean ± SEM. ***, *p* < 0.0001; ****, *p* < 0.00001.

The specificity of the HEK dual hTLR5 sensor for *S. enterica* was determined by testing the top 20 *S. enterica* serovars and non-*Salmonella* bacteria [50]. An MOI 1 and the bacterial exposure time of 4 and 6 h was used, and the cell supernatants were tested for alkaline phosphatase activity. All 20 tested *Salmonella* strains showed positive signals and values were ~8- and 16-fold higher than the negative controls at 4 h and 6 h, respectively (Figure 2a). *E. coli* O157:H¯493-89, a non-motile strain, was used as a negative control and baseline to ensure that observed TLR5 responses were motility-dependent (Figure 2a).

While our system is specifically designed to detect *Salmonella* flagellin, other bacterial flagellins may also be recognized to varying degrees, depending on their amino acid sequence similarities to *Salmonella* flagellin. This study addressed this potential cross-reactivity issue by testing a broad collection of non-*Salmonella* bacterial cultures. We observed positive responses from some of these non-*Salmonella* species (Figure 2b). However, as illustrated in Figure 2a, the highest signal intensity was consistently obtained from *Salmonella* spp., which underscores the preferential recognition of *Salmonella* flagellin over other bacteria for the reliable detection of *Salmonella*. This robust response to *Salmonella* flagellin, compared to other bacterial flagellins, highlights the excellent capacity of the HEK-hTLR5 Reporter Cell-based Sensor to be used effectively and reliably for *Salmonella* detection in food samples.

Compared to *Salmonella* Enteritidis (PT21), most non-*Salmonella* bacteria, including *Hafnia alvei*, *Serratia marcescens*, *Proteus mirabilis*, *Proteus vulgaris*, *Klebsiella pneumoniae*, and a few *E. coli* strains (O157:H7, O26, and O103) showed a 2~3-fold decrease in TLR-5 response. In contrast, two out of three strains—*Citrobacter freundii* and *E. coli* O157:H7—showed higher responses (Figure 2b). The cytotoxic effect of the test strains on HEK dual hTLR5 cells was analyzed by monitoring the release of lactate dehydrogenase (LDH; Figure 2c). In general, cytotoxicity values were below 20% and significantly lower than the positive control (1% Triton X-100 or *L. monocytogenes*), suggesting that the flagella-mediated signal response obtained was largely independent of cellular damage. These results indicate that the HEK dual TLR5 sensor can be used to detect *Salmonella* serovars due to TLR-5 recognizable flagellin expression [46]. Any non-*Salmonella* bacteria that produce a TLR-5 response could be eliminated during selective enrichment and/or IMS separation (see below).

### 3.3. Detection of Live and Stress-Exposed Salmonella Using HEK Dual TLR5 Sensor

**Figure 3:**
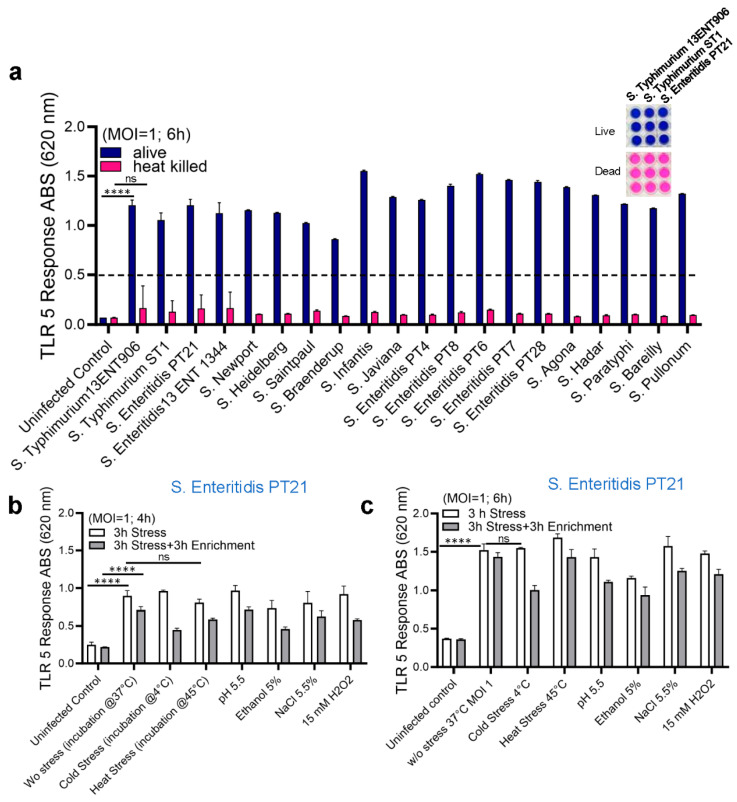
Analysis of the HEK Dual TLR5 Sensor’s Response in the presence of stressors. (**a**) Response to live and dead bacteria: this panel illustrates the HEK dual TLR5 sensor’s response to both live and dead bacteria after 6 h of exposure at a multiplicity of infection (MOI) 1. Panels on the top right depict representative color changes of the HEK dual TLR5 sensors between live (purple) and dead (pink) tested samples. (**b**,**c**) Effects of stressors on TLR5 response: these panels investigate how different stressors affect the TLR5 response of *Salmonella* PT21. Panel (**b**) shows the TLR5 response after 4 h of exposure, while panel (**c**) presents the response after 6 h of exposure, both tested at an MOI of 1. Data in (**a**–**c**) represent the average results from three independent experiments presented as mean ± SEM. ****, *p* < 0.00001.

We next tested if the HEK dual hTLR5 sensor could differentiate live from dead cells since processed food products may carry dead cells. To determine the sensor’s response to living and dead cells, overnight-grown fresh cultures of different *Salmonella* serovars were prepared, and MOI was adjusted to 1. Salmonellae were treated with heat (80 °C for 10 min) and plated on TSA to ensure cells were inactivated (non-viable). AP levels were monitored after 6 h exposure of HEK dual hTLR5 sensor to *Salmonella*. Only the live cells of *S. enterica* serovars showed positive signals (~10-fold increase), while heat-inactivated dead cells did not yield a signal (Figure 3a).

Next, we tested the effects of various stressors on detecting the *S.* Enteritidis PT21 strain that was subjected to multiple stressors, including cold (4 °C), heat (45 °C), acidity (pH 5.5), ethanol (5%, *v*/*v*), NaCl (5.5%), and oxidative stress (H_2_O_2_, 15 mM) for 3 h followed by 3 h resuscitation in TSBYE [36,52]. Bacterial cell suspensions were plated to assess cell viability and cell counts (Table 2) to ensure the viability of *Salmonella* following stress induction and to confirm the desirable MOI before exposure to the HEK dual hTLR5 sensor. All stressors showed positive results in the tested *Salmonella* PT21 strain at both 4 and 6 h, respectively (Figure 3b,c), however, there was a slight but negligible reduction in signal response (Figure 3b,c). These results suggest that the HEK dual hTLR5 sensor can detect viable or stress-exposed *Salmonella* cells.

### 3.4. Detection of Salmonella from Spiked Food Samples Using HEK Dual TLR5 Sensor

**Figure 4:**
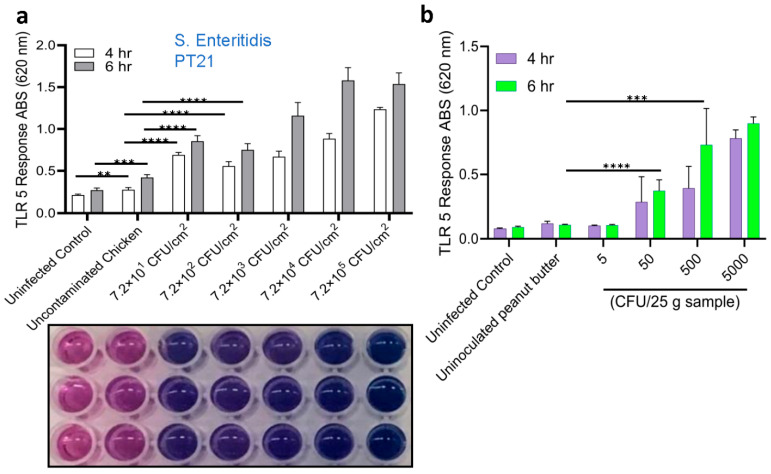
Limit of detection analysis of HEK dual TLR5 sensors to detect *Salmonella* from spiked chicken skin and peanut butter samples. (**a**) TLR 5 sensors’ response after 4 and 6 h incubation of spiked chicken samples. Plate image representative showing color development at 6 h (below). (**b**) TLR 5 sensors’ response after 4 and 6 h incubation of spiked peanut butter samples. Data in (**a**,**b**) represent the average results from three independent experiments presented as mean ± SEM. **, *p* < 0.001; ***, *p* < 0.0001; ****, *p* < 0.00001.

The performance of the HEK dual hTLR5 sensor was validated using artificially spiked food samples. Initially, the LOD for the sensor was determined from spiked chicken and peanut butter samples. Background flora on tested chicken thigh meat samples was 1.92 CFU/cm^2^, but tested negative for *Salmonella*. Chicken thigh meat samples were artificially contaminated with different levels of *S.* Enteritidis PT21 cells (7.2 × 10^5^ CFU/cm^2^, 7.2 × 10^4^ CFU/cm^2^, 7.2 × 10^3^ CFU/cm^2^, 7.2 × 10^2^ CFU/cm^2^, 7.2 × 10^1^ CFU/cm^2^) and incubated overnight in the cold room (4 °C). Skin surfaces were swabbed using Dacron swabs, transferred to tubes containing 1 mL of PBS (pH 7.2), serially diluted, and 100 µL samples were added to HEK dual hTLR5 cells growing in a 96-well cell culture plate and incubated for 4–6 h. Bacterial counts in the inoculums were enumerated to be 4.3 ×10^6^, 3.6 × 10^5^, 2.7 × 10^4^, 3.2 × 10^3^, and 1.0 × 10^3^ CFU/mL, respectively. Relative to uncontaminated meat, *S.* Enteritidis cells from spiked samples at all inoculation levels showed positive signals at 4 h and 6 h. The values were 2.5–6-fold higher (*p* < 0.05) than the uninoculated sample (Figure 4a). Visual examination of color changes in the wells (6 h) corroborates the absorbance readings (Figure 4a, bottom panel). These data indicate that the TLR5 sensor could detect as few as 100 *Salmonella* cells from the chicken samples.

The LOD of the sensor was also determined using peanut butter. No background flora were found in tested peanut butter samples, which were also negative for *Salmonella* (Table 3). Peanut butter (25 g per portion) was spiked with *S.* Enteritidis PT21 to obtain an initial inoculum of about 5, 50, 500, and 5000 CFU/25 g, and enrichment steps followed a shortened USDA-FSIS procedure, as described before, and confirmed via plate CFU counts (Table 3) [50,54]. The initial inoculum containing 50–5 × 10^3^ CFU/25 g showed positive signals, and the incremental values were proportional to the initial inoculum levels. Furthermore, these values significantly differed from the signals obtained from the uninoculated samples or those of peanut butter inoculated with 5 CFU/25 g (Figure 4b). These data indicate that the sensor can detect about 50 *Salmonella* cells (CFU/25 g) (as an initial inoculum) without interference from peanut butter samples.

### 3.5. Validation of Sensor with Spiked Food Samples

Next, we tested the HEK dual hTLR5 sensor with additional spiked food samples (100 CFU/25 g; chicken thigh meats, black pepper, peanut kernel, peanut butter, mayonnaise, peach) where sample preparation and enrichment steps followed a shortened USDA-FSIS procedure [50,54] (Table 3). One hundred microliters of enriched samples were tested for their response to HEK dual hTLR5 cells. Each experiment was repeated five times for all food samples on different days.

**Figure 5:**
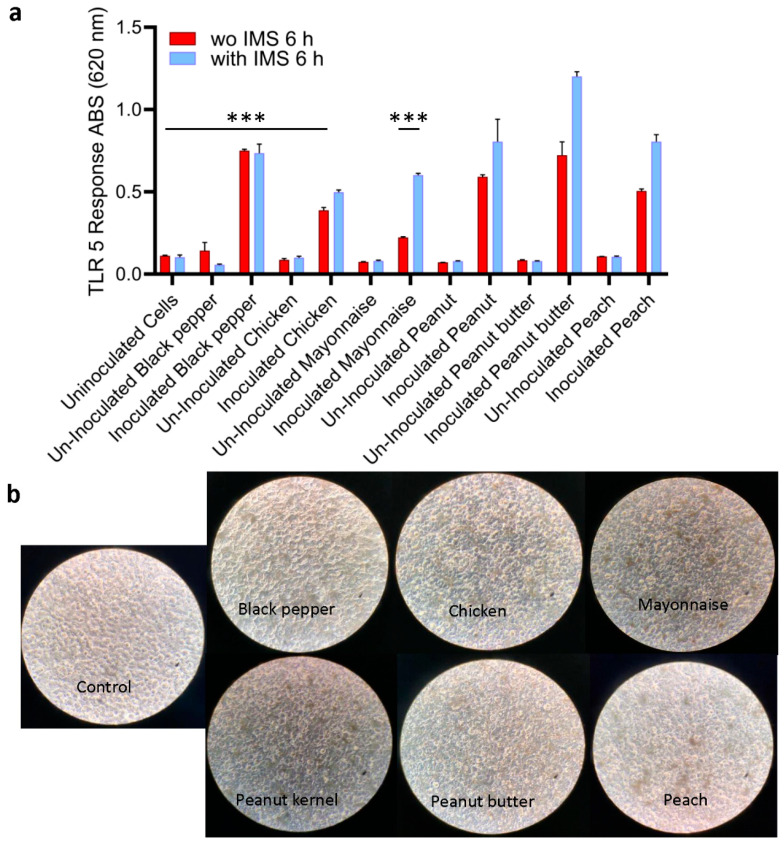
Detection of *Salmonella* using HEKdualTLR 5 sensor from spiked food samples. (**a**) TLR 5 sensor response after 6 h incubation of spiked food samples. Spiked food samples were subjected to 4-h pre-enrichment and 4-h selective enrichment (initial contamination level was ~100 CFU/25 g food sample). *Salmonella* was captured and concentrated using immunomagnetic beads (IMB) in tests with IMS. Data represent the average results from three independent experiments presented as mean ± SEM. ***, *p* < 0.0001 (**b**) Representative microscopic images show an intact cell monolayer treated with bacterial suspension concentrated from all food samples.

All *S.* Enteritidis-inoculated samples, irrespective of food types, produced ~5–10-fold higher signals (*p* < 0.001) than the uninoculated food samples (Figure 5a). Interestingly, the signal from inoculated mayonnaise was lowest among all the food samples tested. Furthermore, microscopic observation of HEK dual hTLR5 cells after exposure to various food samples also did not affect the cell monolayer integrity or cellular morphology (Figure 5b). In parallel, the same set of samples was tested with plate counting (on XLD) methods and PCR for verification. Compared to the TLR5 sensor, PCR could only detect *Salmonella* in peanut kernels, while other spiked food samples post-enrichment tested negative (Table 4). Negative PCR results from inoculated food samples are attributed to the presence of PCR inhibitors. These data suggest that the TLR5 cell sensor can detect as low as 100 CFU/25 g of pre-enriched food samples with a complex food matrix.

Next, we employed immunomagnetic separation (IMS) to concentrate *Salmonella* from enriched food samples before testing with the HEK dual hTLR5 sensor to determine if the signal could be augmented. Before conducting food analysis using the IMS method, we investigated the immunomagnetic bead (IMB)-based capture rate of freshly grown and diluted (8.99 to 1.99 log CFU/mL bacteria) cultures of *S.* Enteritidis by plating. The capture rate was calculated to be >90% for all concentrations, regardless of the bacterial load (Table S1). The application of IMS significantly improved the signal, except for the inoculated black pepper (Figure 5a). The sample-to-result time was estimated to be 15 h.

## 4. Discussion

The risk-based rapid and sensitive detection of *Salmonella* species is highly desirable to protect consumers from food-associated illnesses and promote food safety and biosecurity. Although various detection platforms have been developed in recent years, the traditional culture-based detection methods require 4–7 days to yield results [36,54,55,56], whereas rapid methods still take at least 24–48 h [25,37,57,58,59]. This delay poses a significant inconvenience to the food industry, especially for products with short shelf lives. Additionally, the requirement to store products until microbiological safety is confirmed increases storage costs. Therefore, products often enter the supply chain before test results are available, which is costly and results in hundreds of recalls each year and millions of pounds of food wasted, which is unsustainable for meeting the future food demand [60,61]. This situation creates a high demand for quick, accurate, easy-to-use pathogen detection tools to reduce recalls, minimize food waste and financial losses, and prevent foodborne illnesses. Mammalian cell-based assays are highly promising for functional screening because they can detect viable pathogens in real-time [38]. These assays monitor host–pathogen interactions [39], ensuring that non-pathogenic, non-hazardous, dead, or non-toxic agents do not produce false results.

This study uses a HEK293 cell line expressing TLR-5, and a chromogenic reporter system to detect viable *Salmonella* in a 96-well tissue culture plate. The HEK-dual hTLR5 cell-based functional biosensor construction involves several critical molecular engineering steps to achieve specificity, sensitivity, and robustness for detecting *Salmonella* flagellin. TLR5 is a pattern recognition receptor (PRR) that plays a crucial role in the innate immune system by recognizing bacterial flagellin, a component of the bacterial flagellum. Flagellin is a bacterial protein that polymerizes into the flagellar filament and allows bacteria to be motile. During the bacterial invasion of the host cell, flagellin is recognized by TLR5 and activates innate immune signal transduction cascades [46,62,63]. TLR5 is an innate immune receptor located on the cell surface and consists of an extracellular leucine-rich repeat (LRR), transmembrane, and intracellular domains [64]. TLR5 uses the extracellular domain to recognize flagellin as a pathogen-associated molecular pattern (PAMP). It activates the MyD88-dependent signaling pathway and NF-κB-mediated production of proinflammatory cytokines, and this response can be measured using the chromogenic detection system (Figure 1) [44].

Previous research on TLR5 has demonstrated how this receptor recognizes bacterial flagellin and triggers the innate immune response, providing crucial context for its application in biosensor development [62]. IL-8 (interleukin 8) is a chemokine produced in response to TLR agonists in an NF-κB/AP-1-dependent manner [44]. This feature enables the double readout study of the NF-κB/AP-1 pathway by monitoring the activity of SEAP and Lucia luciferase using a QUANTI-Blue™ Solution (SEAP detection reagent) or QUANTI-Luc™ 4 Lucia/Gaussia (luciferase detection reagent). Thus, researchers may choose the readout depending on laboratory equipment, utilizing a spectrophotometer for SEAP or a luminometer for Lucia luciferase detection according to manufacturers instruction (InvivoGen, San Diego, CA, USA). HEK293 cells are commonly used in biosensing due to their well-characterized genetics, ease of transfection, and robust growth in culture. These cells are particularly suitable for engineering to express receptors like TLR5 because they provide a human cellular context that closely mimics natural physiological conditions [65].

The HEK293 cells are genetically modified to express human TLR5 (hTLR5). This is typically achieved by transfecting the cells with a plasmid containing the hTLR5 gene under the control of a strong promoter [e.g., cytomegalovirus (CMV) promoter] to ensure high levels of expression. The successful expression of hTLR5 on the surface of HEK293 cells allows these cells to respond specifically to flagellin by activating downstream signaling pathways.

The biosensor’s specificity is primarily derived from the selective binding of TLR5 to *Salmonella* flagellin. While TLR5 can recognize flagellin from other bacterial species, the structural differences in flagellins contribute to varying degrees of TLR5 activation. The optimization process includes testing different *Salmonella* serovars and comparing the response to non-*Salmonella* flagellins to ensure that the sensor preferentially detects *Salmonella* [64].

This cell-based bio-detection system is optimized to amplify the signal generated by TLR5 activation. This involves fine-tuning the expression levels of TLR5 and the reporters, as well as optimizing the cell culture conditions (e.g., cell density and media composition) to maximize sensitivity and minimize background noise. A dual reporter system enhances the signal-to-noise ratio and improves the biosensor’s overall robustness. By elaborating on these molecular engineering steps above, we aim to provide a clearer understanding of how the HEK-dual hTLR5 cell-based functional biosensor was constructed and optimized for the specific detection of *Salmonella* flagellin.

The design of the HEK-dual hTLR5 cell-based functional biosensor in this study primarily focused on single-use applications to ensure optimal sensitivity and specificity. Single-use biosensors are advantageous as they minimize potential contamination risks and ensure that each assay operates under ideal conditions, free from any residual effects of prior use. However, the concept of substrate reuse is increasingly relevant in practical applications, mainly where cost-effectiveness and sustainability are key considerations. Reusing substrates could reduce operational costs and environmental impact, making the technology more accessible for widespread use in food safety monitoring. In conclusion, while the HEK-dual hTLR5 cell-based biosensor was designed for single-use, exploring substrate reuse presents an exciting avenue for future research, with potential benefits for both cost-efficiency and environmental sustainability in biosensor applications.

To test our hypothesis that the HEK-TLR-5 sensor can detect viable *Salmonella* selectively, we first determined the motility of different types of common foodborne bacterial pathogens (*Salmonella* Enteritidis PT21, *E. coli* O157:H7, and *Listeria monocytogenes*). The motility test (plating results performed on agar surface) and the signals obtained from the HEK dual hTLR5 sensor correlated as *Salmonella* was found to be the most motile bacteria with the highest swimming ability, and the sensor with a flagella recognition feature showed the highest response when tested with *Salmonella* Enteritidis PT21. Smith et al. [63] reported that TLR5 recognizes a conserved flagellin region essential for bacterial motility across different Gram-negative bacteria. The research highlights that while TLR5 primarily responds to flagellin from *Salmonella,* it also exhibits cross-reactivity with flagellins from other Gram-negative bacteria, such as *E. coli* and *Pseudomonas aeruginosa* [63]. Other research [66] has highlighted that TLR5 can detect flagellin from various bacterial pathogens, including *Legionella pneumophila* and *Listeria monocytogenes*, although with varying degrees of effectiveness. The study demonstrates the potential for cross-reactivity, which can be both an advantage for broad pathogen detection and a challenge for specificity [67]. Building on the insights from these studies, our sensor generates robust signals for *Salmonella* species. Furthermore, we have also implemented immunomagnetic separation as a preprocessing step. By employing anti-*Salmonella* Dynabeads for selective isolation from samples, followed by cell-based sensing, we ensured the system’s specificity for *Salmonella* detection.

Furthermore, flagellar expression is critical for obtaining positive signals. We verified relative flagellar expression in test strains by measuring their motility on agar plates containing variable amounts of agar (0.3–1.0%; *w*/*v*) [51]. Swimming motility is the movement in liquid or low-viscosity conditions (up to 0.3% agar concentration). Unlike swarming motility, swimming motility requires a functional flagellum, but neither quorum sensing systems nor biosurfactants [51,53]. Plates with up to 0.3% agar concentration are called ‘swim plates’ because highly motile flagellum-dependent bacteria can swim through the porous medium, leading to a large area of colonization [53]. Plates with agar concentration higher than 0.3% are known as ‘swarm plates’ because the reduced pore size prevents swimming through the agar, and extensive colonization of the plate occurs only through bacterial migration across the surface.

Specificity testing of the HEK-hTLR5 Reporter Cell-Based Sensor showed a high response (6-fold increase; Figure 2b) with all the top 20 *Salmonella* serovars tested. Testing with the non-*Salmonella* organisms suggested minimal signals with several common foodborne bacterial pathogens such as Enterohemorrhagic *Escherichia coli* (EHEC) strains, *H. alvei*, *S. marcescens*, *K. pneumoniae*, and *Proteus vulgaris,* suggesting their flagellin molecules may be poorly recognized by TLR5 [46]. However, two out of three *Citrobacter* strains showed cross-reactivity, which may be attributed to high TLR-5 recognizable flagellin expression. More importantly, when tested with food samples, the sensor was specific as it showed no response to natural microflora or several uninoculated food samples. Black pepper, chicken, mayonnaise, peanut kernels, peanut butter, and peaches were chosen because these products were linked with *Salmonella* outbreaks, and some are consumed without heat treatment. In addition, they have high protein, fat, and carbohydrate contents, which may cause interferences. Our data demonstrated that the tested uninoculated food samples had minimal or no interference with sensor response, but *Salmonella*-inoculated samples showed a strong response. Surprisingly, PCR assay with inoculated food samples gave negative PCR results (except peanut kernels) (Table 3), and the negative PCR results are attributed to the presence of PCR inhibitors in these samples [67].

The sensitivity of the cell-based sensor in pure culture suggested that an MOI of 0.1 in the 10^5^ seeded HEK-hTLR5 reporter cells on a 96-well plate elicited a positive signal (Figure 1d). These data indicate that the LOD in pure culture is ~10^5^ CFU/mL (10^4^ CFU/well in 100 µL). Our results are comparable with the LOD of the recently developed Next-Generation Sequencing Assay for direct detection of *Salmonella* [68]. Further testing of LOD in spiked food samples suggested the LOD is as low as 50–100 CFU/25 g of food samples, which has the potential to outperform other molecular detection methods such as ELISA or PCR [59]. Our results are comparable with previous research showing an IMS-PCR procedure designed to isolate and detect *S. enterica* in vegetable salad, egg, and pork meat, with a detection sensitivity of 10^2^ CFU/mL [69]. However, one significant advantage of the HEK-hTLR5 cell-based sensor is its ability to selectively detect viable *Salmonella* and not respond to dead cells (Figure 3a). Additionally, although the sensitivity of the sensor may be high, accurate results may not be obtained due to the low level of pathogen survival in stressed food conditions.

The use of HEK cells has some limitations that can influence the reproducibility and sensitivity of the results. One critical factor is HEK cell density; maintaining an optimal cell density is essential for consistent assay performance, as both under-confluent and over-confluent cultures can lead to variability in the sensor’s response. Additionally, the time since passaging is important—cells that have undergone too many passages may exhibit altered responses due to physiological changes. In addition, from the very first day of obtaining the cell lines, we paid meticulous attention to the passaging process. We prepared many stocks from the original cell line from the first and second passages to ensure consistency and reduce the potential for variation over time. This careful handling and preparation of the cell stocks helped maintain the integrity and performance of the HEK-hTLR5 Reporter Cell-based Sensor throughout our study. Another crucial parameter is the MOI, which refers to the ratio of bacteria to host cells in the assay. To minimize the effects of these limitations, we standardized the MOI at 1 in our experiments. This MOI 1 was chosen to ensure that the interaction between bacterial flagellin and TLR5 is effective and consistent, enhancing the assay’s reproducibility. Furthermore, we used a consistent experimental setup throughout the study to prevent the assay from being affected by other physical conditions, such as variations in media composition, pH, and temperature. This included using identical culture media, controlled incubation conditions, and standardized protocols for cell handling and bacterial inoculation. These measures were implemented to mitigate the potential impacts of these variables on the assay’s performance.

The HEK-hTLR5 reporter cell-based sensor was validated further, and tested positive for the detection of *Salmonella* in stressed conditions such as cold (4 °C), heat (45 °C), acid (pH 5.5), ethanol (5%), NaCl (5.5%), and oxidative stress (15 mmol H_2_O_2_). A brief sample enrichment step allowed the resuscitation of stressed or injured cells before detection.

Combining enrichment steps with IMS improved the TLR-5 sensor sensitivity in certain food products, such as mayonnaise. It yielded comparable results with other studies that used IMS tandem fluorescent probes based on quantum dots-antibody (QDs-Ab) for *Salmonella* testing [70]. However, the sensitivity of this assay was lower than our cell-based sensor (500 CFU/mL). Similarly, a protein chip immunosensor was exploited to detect *Listeria* spp. by lowering the detection limit to 2.5 CFU/mL with the pre-enrichment culture of IMS [71]. While IMS can be evaluated as a potential pretreatment tool for the rapid isolation and enrichment of many foodborne pathogens in food, the bacterial surface antigens from dead cells could still bind the capture antibody. Therefore, our TLR-5 cell-based sensor combined with the IMS method is significantly more advantageous when viable pathogens are sought. Thus, developing cell-based assays is critical for functional screening and eliminating false-negative results from dead, non-hazardous, or non-pathogenic bacteria.

In conclusion, our two-step method (enrichment and TLR-5 sensor), with a significantly lower detection time of 15 h from sample to result, was found to be highly specific for the detection of live salmonellae in complex food matrixes in the presence of stressors with a sensitivity of 50 CFU/25 g of food. Therefore, the TLR-5 sensor could be used as a risk-based screening tool for viable *Salmonella*.

## Figures and Tables

**Table 1:** List of bacterial strains used in the study from our culture collection.

*Salmonella enterica* Serovars	Non-*Salmonella* Cultures
Enteritidis PT4	*Escherichia coli* O157:H¯493-89 (flagella negative control)
Enteritidis PT8	*E. coli* O26:H11 00971
Enteritidis PT6	*E. coli* O103:H2 90-3128
Enteritidis PT7	*E. coli* O26:H11 05-6544
Enteritidis PT28	*E. coli* O103:H11 SJ12
Enteritidis PT21	*E. coli* O157:H7 204P
Enteritidis 13ENT1344	*E. coli* O157:H7 505B
Typhimurium 13ENT906	*E. coli* O157:H7 ATCC 43895
Typhimurium ST1	*E. coli* O157:H7 ATCC 43890
Agona 12ENT1356	*Citrobacter freundii* ATCC 3624
Hadar 13ENT979	*Citrobacter freundii* ATCC43864
Paratyphi 11J85	*Citrobacter freundii* ATCC8090
Bareilly 12ENT1164	*Hafnia alvei*
Pullorum DUP-PVUII 1006	*Proteus vulgaris* DUP-10086
Newport 13ENT1060	*Proteus mirabilis* B-3402
Heidelberg 18ENT1418	*Serratia marcescens* ATCC 8100
Saintpaul 13ENT1045	*S. marcescens* B-2544
Bradenderup 12ENT1138	*S. marcescens* ATCC 43862
Infantis 13ENT866	*Klebsiella pneumoniae* B-41958
Javiana 13ENT86F	*Listeria monocytogenes* 104033S

**Table 2:** Effect of 3-h environmental stress on *Salmonella* Enteritidis PT21 viability and growth.

	Log _10_ (CFU/mL) ± SEM (N = 3)						
	No Stress	Cold Stress	Heat Stress	Low pH	Ethanol	NaCL	H_2_O_2_
3-h stress	9.86 ± 0.19	9.38 ± 0.21	9.35 ± 0.11	9.67 ± 0.14	9.60 ± 0.22	9.24 ± 0.28	9.33 ± 0.21
3-h stress + 3-h enrichment	10.49 ± 0.22	10.44 ± 0.24	10.01 ± 0.21	10.21 ± 0.24	9.69 ± 0.29	10.33 ± 0.19	10.65 ± 0.24

**Table 3:** Recovery of *Salmonella* Enteritidis PT21 from contaminated peanut butter at each step of sample enrichment. TSA, Tryptic soy agar; XLD, Xylose lysine deoxycholate.

		Log _10_ (CFU/mL) ± SEM (N = 3)		
Inoculation Level (CFU/25 g Sample)	Pre-Enriched		Enriched	
	TSA	XLD	TSA	XLD
0	No growth	No growth	No growth	No growth
~5	No growth	No growth	2.86 ± 0.21	3.09 ± 0.18
~50	2.68 ± 0.17	2.51 ± 0.11	4.06 ± 0.14	4.18 ± 0.19
~500	3.94 ± 0.21	3.85 ± 0.19	6.32 ± 0.22	6.39 ± 0.24
~500	4.35 ± 0.13	4.44 ± 0.18	6.60 ± 0.23	6.42 ± 0.14

**Table 4:** Recovery of *Salmonella* Enteritidis PT21 from spiked (~100 CFU/25 g, except peach (~100 CFU/100 g) food samples at each step of sample enrichment and concentration.

	Log _10_ (CFU/mL) ± SEM (N = 3)							
Food Sample	After Selective Enrichment		Without IMS		IMS		Counts (CFU/100 µL)	PCR (*invA* 796 bp)
	TSA	XLD	TSA	XLD	TSA	XLD	XLD	
Black pepper (U)	0	0	0	0	0	0	0	-
Black pepper (I)	3.91 ± 0.18	4.04 ± 0.11	3.87 ± 0.14	4.05 ± 0.21	3.98 ± 0.08	4.07 ± 0.14	3.07 ± 0.11	-
Chicken (U)	0	0	0	0	0	0	0	-
Mayonnaise (U)	0	0	0	0	0	0	0	-
Mayonnaise (I)	3.94 ± 0.22	4.02 ± 0.14	3.72 ± 0.18	3.94 ± 0.12	3.72 ± 0.21	3.92 ± 0.18	2.92 ± 0.13	-
Peanut kernel(U)	0	0	0	0	0	0	0	-
Peanut kernel (I)	3.92 ± 0.26	4.09 ± 0.19	4.26 ± 0.22	4.29 ± 0.18	4.07 ± 0.14	4.13 ± 0.19	3.13 ± 0.18	+
Peanut butter (U)	0	0	0	0	0	0	0	-
Peanut butter (I)	5.53 ± 0.24	5.18 ± 0.18	5.36 ± 0.25	5.46 ± 0.28	5.39 ± 0.22	5.47 ± 0.24	4.47 ± 0.16	-
Peach (U)	0	0	0	0	0	0	0	-
Peach (I)	3.71 ± 0.14	3.88 ± 0.16	3.72 ± 0.12	4.08 ± 0.19	3.91 ± 0.16	4.33 ± 0.14	3.33 ± 0.11	-

U, uninoculated; I, inoculated; IMS, immunomagnetic separation.

## Data Availability

All data are presented in the manuscript and Supplementary Files.

## Supplementary Materials

The following supporting information can be downloaded at: https://www.mdpi.com/article/10.3390/bios14090444/s1, Figure S1: analysis of bacterial motility on agar plates; Table S1: performance of anti-salmonella dynabeads.
