# Supplementary material for: Salmonella Detection in Food Using a HEK-hTLR5 Reporter Cell-Based Sensor
Source: Biosensors (Basel). 2024 Sep 18;14(9):444. doi: 10.3390/bios14090444 (PMC11430776; doi:10.3390/bios14090444)
Supplement: Supplementary file 1 [file biosensors-14-00444-s001.zip › biosensors-3172697-supplementary.pdf]

biosensors

MDPI

Supplementary materials

Salmonella Detection in Food Using a HEK-hTLR5 Reporter Cell-Based Sensor

Esma Eser [1,2], Victoria A. Felton [3], Rishi Drolia [1,3,4,5]* and Arun K. Bhunia [1,4,6,]*

[1] Molecular Food Microbiology Laboratory, Department of Food Science, Purdue University, West Lafayette, IN 47907, USA; esmaeser@comu.edu.tr
[2] Department of Food Engineering, Faculty of Engineering, Canakkale Onsekiz Mart University,
Canakkale 17100, Turkey
[3] Molecular and Cellular Microbiology Laboratory, Department of Biological Science, Old Dominion
University, Norfolk, VA 23529, USA; vfelt001@odu.edu
[4] Purdue Institute of Inflammation, Immunology and Infectious Disease, Purdue University, West Lafayette, IN 47907, USA
[5] Center for Bioelectronics, Old Dominion University, Norfolk, VA 23508, USA
[6] Department of Comparative Pathobiology, Purdue University, West Lafayette, IN 47907, USA
* Correspondence: rdrolia@odu.edu (R.D.); bhunia@purdue.edu (A.K.B.)

biosensors

MDPI

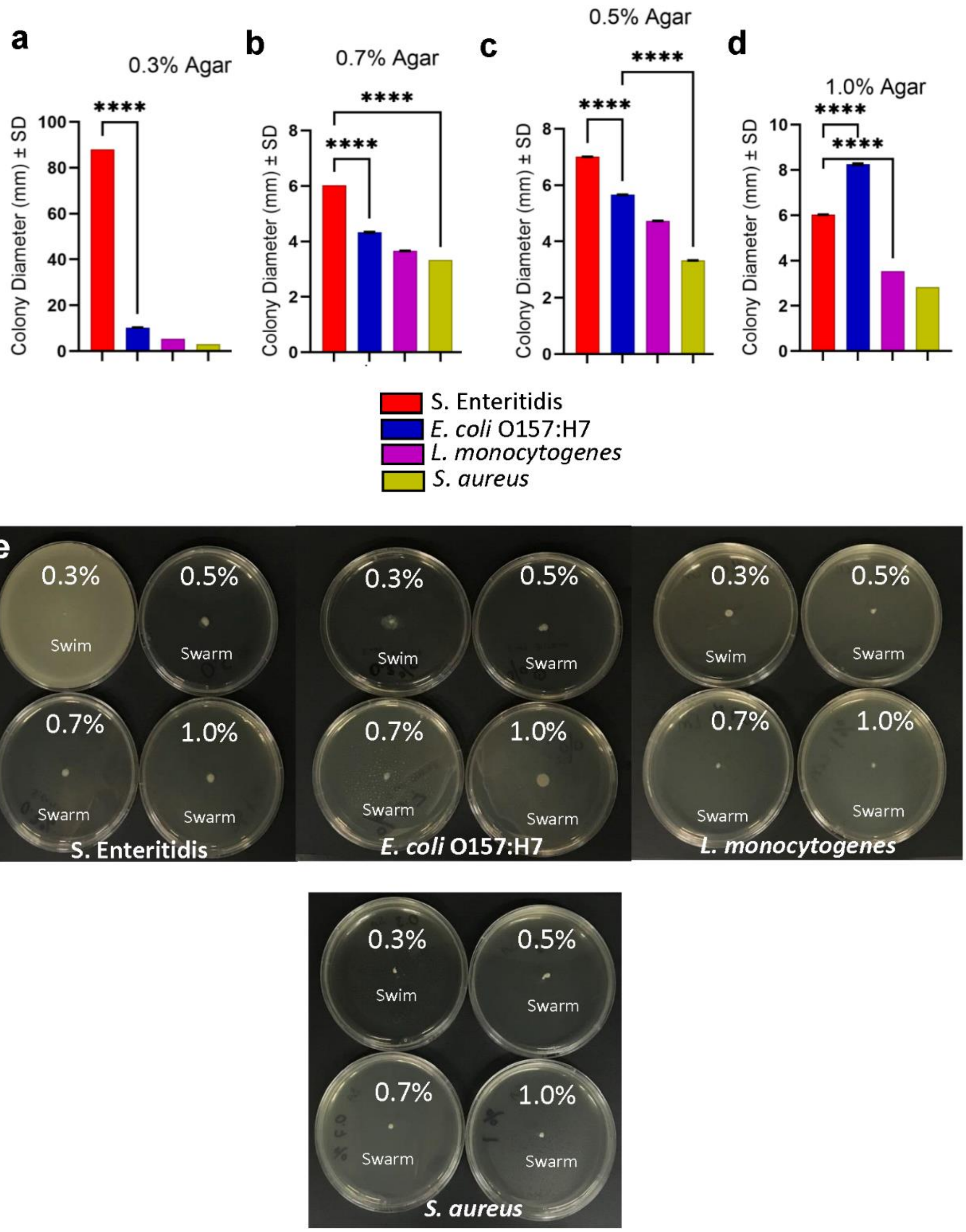

Figure S1. Analysis of Bacterial Motility on Agar Plates. (**a-d**) Graphs representing Average Colony diameter (mean ± SEM). (**e**) Representative motility agar plate images.

biosensors

MDPI

Table S1. Performance of Anti-Salmonella Dynabeads.

	Average Culture Density (Log_{10} CFU/mL)							
**Before IMS**	8.99	7.99	6.99	5.99	4.99	3.99	2.99	1.99
**After IMS**	8.48	7.32	6.60	5.88	4.86	3.78	2.89	1.88
**Capture efficiency (%)**	94.25	91.52	94.39	98.00	97.31	94.63	96.48	94.00

IMS, Immunomagnetic separation
